# Defining and sparing sexual function-related organs at risk for rectal cancer radiotherapy

**DOI:** 10.2340/1651-226X.2025.44011

**Published:** 2025-08-28

**Authors:** Camilla J.S. Kronborg, Dennis T. Arp, Rana Bahij, Susan B.N. Biancardo, Laura V. Diness, Kenni H. Engstrøm, Lars U. Fokdal, Bodil G. Pedersen, Birgitte Havelund, Christian A. Hvid, Kirsten L. Jakobsen, Kathrin Kirchheiner, Christina M. Lutz, Lars Nyvang, Birthe T. Oggesen, Stine E. Pedersen, Laurids Ø. Poulsen, Heidi S. Rønde, Lise K. Schou, Eva Serup-Hansen, Johanne H. Steffensen, Jimmi Søndergaard, Joanna E. Szpejewska, Henrik D. Nissen

**Affiliations:** a Danish Centre for Particle Therapy, Aarhus University Hospital, Aarhus, Denmark; b Department of Clinical Medicine, Aarhus University, Aarhus, Denmark; c Department of Oncology, Aalborg University Hospital, Aalborg, Denmark; d Department of Oncology, Odense University Hospital, Odense, Denmark; e Department of Oncology, Copenhagen University Hospital – Herlev and Gentofte, Herlev, Denmark; f Department of Oncology, University Hospital of Southern Denmark, Lillebaelt Hospital, Vejle, Denmark; g Department of Radiology, Aarhus University Hospital, Aarhus, Denmark; h Department of Oncology, Aarhus University Hospital, Aarhus, Denmark; i Department of Oncology, Zealand University Hospital, Næstved, Denmark; j Department Radiation Oncology, Medical University Vienna / General Hospital Vienna, Vienna, Austria; k Department of Surgery, Copenhagen University Hospital, Herlev and Gentofte Hospital, Herlev, Denmark; l Department of Clinical Medicine, University of Copenhagen, Copenhagen, Denmark

**Keywords:** Sexual health, sexual dysfunction, intensity-modulated radiotherapy, rectal cancer, multidisciplinary

## Abstract

**Background and purpose:**

Sexual dysfunction is a common consequence of pelvic radiotherapy, influenced by psychological, physical, social, and relational factors. Research has focused on vaginal dose and stenosis in females and penile bulb dose and erectile dysfunction in males, with limited attention to domains, such as arousal, desire, and satisfaction. In the Danish Colorectal Cancer Radiotherapy Group, we aimed to: (1) Develop an atlas of sexual function-related organs at risk and (2) Evaluate if these organs at risk could be spared without compromising target coverage in rectal cancer radiotherapy planning.

**Patient/material and methods:**

A multidisciplinary approach was adopted, involving oncology, physics, psychology, surgery, and radiology. MRI-based anatomical definitions were established, and an atlas was created for both males and females, including inferior hypogastric plexus, pudendal vessels/Alcock’s canal, neurovascular bundle, penile bulb, vagina, paracolpium, and bulboclitoris. For comparative planning standard and sexual function-sparing plans were created for each patient.

**Results:**

A national consensus atlas for sexual function-related organs at risk was developed. Standard plans (*n* = 15) and sexual function-sparing plans (*n* = 15) for seven males and eight females were compared. Sparing of pudendal vessels and bulboclitoris was feasible without compromising the standard plan. For sexual function-related organs at risk in or close to the target, D2% could often be improved.

**Interpretation:**

Our consensus-based delineation and planning demonstrate that radiation dose to many sexual function-related organs at risk can be spared or optimized without compromising target coverage or dose to standard organs at risk. Future work includes implementing patient-reported outcomes and integrating these new organs at risk into standard radiotherapy planning.

## Introduction

Sexual dysfunction is a well-documented consequence of pelvic radiotherapy (RT), resulting from a complex interplay of psychological, physical, social, and relational factors. Despite the clinical importance, the term ‘sexual dysfunction’ is inconsistently defined across studies, contributing to significant variation in reported prevalence following treatment for colorectal cancer – ranging from 5% to 90% [1–3].

Sexual health in rectal cancer patients is influenced not only by the cancer treatment itself but also by secondary effects such as the presence of a stoma, with changes in body image as well as bowel dysfunction, including fecal incontinence [4, 5].

These factors interfere with multiple domains of sexual function, and patients with rectal cancer are reported to be less sexually active than healthy controls. In male patients, erectile dysfunction rates post-treatment range widely from 11% to 90% [2]. Female patients with rectal cancer report higher rates of sexual inactivity compared to women treated for colon cancer, with dyspareunia and vaginal dryness as the most common complaints [6].

In general, the risk of sexual dysfunction is higher after treatment for rectal cancer compared to colon cancer. The contributors, besides surgery, are RT and the presence of a stoma, which have both been identified as independent risk factors for sexual dysfunction in both males and females. Additionally, body image concerns are prominent among patients with a stoma – 40% of women report feeling less feminine and 34% report feeling less attractive, which further compounds sexual distress and dissatisfaction [6, 7].

Although several validated questionnaires and patient-reported outcome measures (PROMs) are available to assess sexual dysfunction, their systematic use in clinical practice remains limited. This contributes to the under-recognition and under-treatment of sexual health issues in patients undergoing pelvic RT [8].

Across studies, RT has been found to be an independent predictor of long-term sexual dysfunction [9]. However, research on dose-volume effects on sexual function-related organs at risk (SF-OARs) has been limited in scope, focusing primarily on vaginal dose and stenosis in female patients [10] and on the penile bulb and erectile dysfunction in male patients [11]. Other crucial domains such as arousal, desire, and sexual satisfaction remain understudied, in part due to the lack of consensus on relevant anatomical targets for potential dose-sparing strategies.

To address this gap, the Danish Colorectal Cancer Radiotherapy Group (DCCG-RT) initiated a multidisciplinary project with two primary aims: (1) to develop a comprehensive atlas of SF-OARs relevant to sexual dysfunction in both male and female patients and (2) to evaluate which of these structures could be spared during rectal cancer radiotherapy planning without compromising target coverage or dose constraints for standard organs at risk.

## Patients/material and methods

### Study design and participants

This study was conducted within the DCCG-RT as a national, multidisciplinary initiative. The working group consisted of clinical oncologists and medical physicists from seven treatment centers across Denmark, as well as a psychologist specializing in sexology and PRO, a colorectal surgeon with expertise in late effects, and a radiologist with advanced knowledge of pelvic MRI.

In the first step, a female and a male MRI-based atlas were developed. In the next step, patients from each center were retrospectively selected for radiotherapy planning analysis, and SF-OARs were delineated. All patients fulfilled national standard indications for neoadjuvant radiotherapy for rectal cancer.

### Atlas development

The atlas development process was initiated during the first multidisciplinary meeting, where pelvic MRI images were reviewed to identify anatomical structures involved in sexual function. A draft list of SF-OARs was generated for both male (M) and female (F) patients. These included for both inferior hypogastric plexus and pudendal vessels and nerves (within Alcock’s canal) and specifically the neurovascular bundle and penile bulb for male patients and the vagina, paracolpium, and the bulboclitoris for female patients, including the crura of the clitoris. This draft atlas was then circulated among all participating centers for review. Other structures such as penile corpus cavernosum and external female genitalia were not included as they have been described elsewhere [11, 12].

During a subsequent 2-day workshop, all proposed SF-OARs were delineated in consensus by the group using standard imaging datasets. Delineation and identification of SF-OAR were done on the MRI acquired in treatment position, and adaptation to CT anatomy was done only if necessary. Throughout the process, SF-OARs were reviewed by a senior radiologist with pelvic MRI expertise. After the 2-day workshop, additional cases were delineated at each center – a minimum of one male and one female per center. These were then discussed and reviewed in follow-up online meetings, leading to refinement and final consensus on both the SF-OAR atlas and a descriptive table.

### Imaging and radiotherapy planning

For RT planning, standard imaging included CT scans with contrast and 2–3 mm slice thickness and MRIs with T2-weighted axial and sagittal views. One center used MR-angio instead of CT contrast, and one center used a MR-only workflow with synthetic CTs. MRIs and CTs were acquired in treatment position and fused based on bony anatomy.

All treatment plans adhered to national guidelines and were based on intensity-modulated radiation therapy (IMRT) or volumetric-modulated arc therapy (VMAT) planning. Elective target volumes included presacral, mesorectal, and lateral pelvic lymph node regions. A total dose of 50.4 Gy in 28 fractions was prescribed to all target volumes.

National standard optimization criteria and constraints are described in the supplementary material. Plans were created by six different physicists, all with at least 8 years of clinical experience.

### Plan comparison

Fifteen patients were selected for plan comparison, with generation of two dose plans per patient, done at each center. First, a standard plan fulfilling criteria according to national guidelines was created; next, a SF-OAR-sparing plan was created. The aim was to achieve as much sparing of SF-OAR as possible while maintaining the quality of the original treatment plan, that is, without clinically relevant increase in any standard OAR dose or decrease in target coverage. The penile bulb could be included in the primary optimization but was further optimized in the SF-OAR plans.

Dose metrics for all delineated SF-OARs, as well as target coverage and standard OARs, were extracted and compared between the two plan types. For this explorative analysis, we used several dose metrics for each SF-OAR, V30Gy (%), V40Gy (%), and D2% (Gy). For target coverage and other OARs, we evaluated standard optimization criteria and constraints.

The Wilcoxon signed-rank test with exact *p*-value calculation was used to assess statistically significant differences in dose parameters between standard and SF-OAR-sparing plans. The *Stata statistical package version 18.5* was used for analyses.

## Results

**Figure 1:**
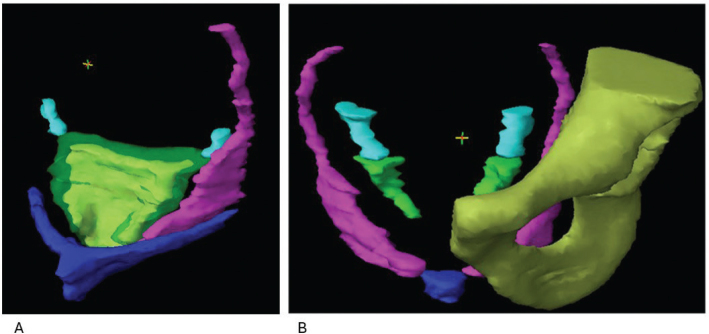
3D visualization of SF-OAR for the female (F) and male (M) pelvis. A (F). Visualization of relations of pudendal vessels/Alcock´s canal (left) (magenta), the inferior hypogastric plexus (cyan), the vagina (yellow) encompassed by the paracolpium (transparent green), and the bulboclitoris including crura (blue). B (M). Visualization pudendal vessels/Alcock´s canal (magenta) and its relation to bone, and the inferior hypogastric plexus (cyan), where in converges with the neurovascular bundle (green). The penile bulb (blue) is visualized where the anterior part of the pudendal vessels end.

A national consensus atlas for SF-OAR (M and F) was developed. In Figure 1, a 3D visualization of F and M SF-OAR is presented. In the supplementary material, axial MR images of F and M SF-OAR are provided, as well as a descriptive table. The atlas included the following structures: shared across M and F: Inferior hypogastric plexus, pudendal vessels, and nerves (Alcock’s canal). M-specific: neurovascular bundle, penile bulb, and F-specific: vagina, paracolpium, and bulboclitoris including crura.

Baseline characteristics can be seen in Table 1, reflecting national guidelines; most patients had T3 or T4 tumors with low- or mid-rectal localization. Standard plans (*n* = 15) and SF-OAR sparing plans (*n* = 15) for seven males and eight females were compared (Table 2).

**Table 1:** Baseline characteristics.

Baseline characteristics	
**Gender, male/female (*n*)**	7/8
**Age, years (median, range)**	67 (50–82)
**TNM**	
**T-stage**	
1	1
2	0
3	9
4	5
**N-stage**	
0	6
1–2	9
**Rectal localization (*n*)**	
Low (0–5 cm)	8
Mid (> 5–10 cm)	6
High (> 10 cm)	1
**Radiation dose (*n*)**	
50.4 Gy/28 fractions	15
**Technique (*n*)**	
VMAT 1–3 arc	13
IMRT	2

VMAT: volumetric-modulated arc therapy; IMRT: intensity-modulated radiation therapy.

**Table 2:** Dose-volume parameters for target, standard OAR, and SF-OAR for both standard and SF-OAR plannings.

	Standard plan *n* = 15 Median (IQR)	SF-OAR-sparing *n* = 15 Median (IQR)	*p*-value
**Standard parameters – target and OAR**			
CTV-T V95% (%)	100 (100–100)	100 (100–100)	1
PTV-T V95% (%)	99.98 (99.7–100)	99.9 (99.8–100)	0.94
PTV-T 105% (%)	0.0 (0.0–0.0)	0.0 (0.0–0.1)	0.063
PTV-E V95% (%)	99.4 (98.6–99.9)	99.3 (98.9–99.9)	0.98
PTV-E 105% (%)	0.0 (0.0–0.08)	0.07 (0–0.37)	0.08
Bowel Bag V45Gy (cc)	239 (120–468)	249 (122–443)	0.99
Bowel Bag V30Gy (cc)	439 (228–700)	445 (210–660)	0.07
Bladder V50Gy (%)	5.1 (0.5–9.4)	4.4 (1–8.5)	0.94
Bladder V35Gy (%)	33.7 (22–42.8)	32.6 (22.4–40.8)	0.038
Sacral bone V50Gy (%)	5.6 (2.9–8.7)	4.4 (3.9–7.1)	0.92
Femoral heads V50Gy (%)	0.0 (0.0–0.0)	0.0 (0.0–0.0)	1
**Sexual function – OAR common**			
Plexus hypogastricus inferius V30Gy (%)	100 (100–100)	100 (100–100)	1
Plexus hypogastricus inferius V40Gy (%)	100 (100–100)	100 (100–100)	1
Plexus hypogastricus inferius D2% (Gy)	52 (51.8–52.3)	50.9 (50.1–51.7)	0.0029
Pudendal artery and nerve + Alcocks canal V30Gy (%)	90.6 (60.2–98.6)	67.4 (41.4–93.3)	0.0001
Pudendal artery and nerve + Alcocks canal V40Gy (%)	45.4 (38.2–72.5)	38.5 (26.3–56.0)	0.0001
Pudendal artery and nerve + Alcocks canal D2% (Gy)	51.3 (50.8–51.5)	50.1 (49.8–51.0)	0.021
**Sexual function – OAR male, *n* = 7**			
Neurovascular bundle V30Gy (%)	100 (100–100)	100 (99.8–100)	0.5
Neurovascular bundle V40Gy (%)	100 (98–100)	100 (97.7–100)	0.5
Neurovascular bundle D2% (Gy)	51.8 (51.3–52.1)	50.8 (50.1–51.3)	0.03
Penile bulb V30Gy (%)	0.0 (0.0–20.4)	0.0 (0.0–8.7)	0.25
Penile bulb V40Gy (%)	0.0 (0.0–10.8)	0.0 (0.0–3.8)	0.5
Penile bulb D2% (Gy)	26.1 (4.2–50)	10.14 (4.3–45.8)	0.063
**Sexual function – OAR female, *n* = 8**			
Vagina V30Gy (%)	100 (100–100)	100 (97.9–100)	0.25
Vagina V40Gy (%)	100 (99.5–100)	100 (92.8–100)	0.25
Vagina D2% (Gy)	51.0 (50.8–51.5)	50.9 (50.0–51.4)	0.46
Paracolpium V30Gy (%)	100 (100–100)	100 (96.3–100)	0.25
Paracolpium V40Gy (%)	100 (96.6–100)	99.7 (90.5–100)	0.13
Paracolpium D2% (Gy)	51.8 (51.1–52.0)	50.8 (50.5–51.6)	0.15
Bulboclitoris V30Gy (%)	63.6 (20.3–71.0)	25.2 (3.1–54.2)	0.015
Bulboclitoris V40Gy (%)	13.7 (0.86–37.0)	5.1 (0.1–23.6)	0.031
Bulboclitoris D2% (Gy)	46.0 (35.4–50.5)	41.6 (28.0–50.7)	0.13

SF-OAR: sexual function-related organs at risk; IQR: interquartile range.

All dose plans, including both standard and SF-OAR-sparing plans, met the predefined national criteria for target volume coverage. CTV-Tumor (T) coverage was optimal in all patients, with 100% of the volume receiving at least 95% of the prescribed dose. PTV-elective (E) and PTV-T coverage were also similar in both plans. The proportion of PTV-T receiving more than 105% of the dose remained low in both groups, although a slight, non-significant increase was seen in SF-OAR-sparing plans (*p* = 0.063) (Table 2).

Dose to standard OAR showed only minor variations when comparing the two plan types. Bowel bag exposure, assessed by V45Gy and V30Gy, showed no significant difference between the two planning approaches (*p* = 0.99 and *p* = 0.07, respectively). Bladder dose V35Gy was slightly lower in the SF-OAR-sparing plans (median 32.6% vs. 33.7%, *p* = 0.038) reflecting vicinity to SF-OAR. No differences were found for femoral heads and sacral bone (Table 2). Supplementary Figure 1 shows dose-volume histograms for representative examples of bowel and bladder dose for standard and SF-OAR sparing plans.

**Figure 2:**
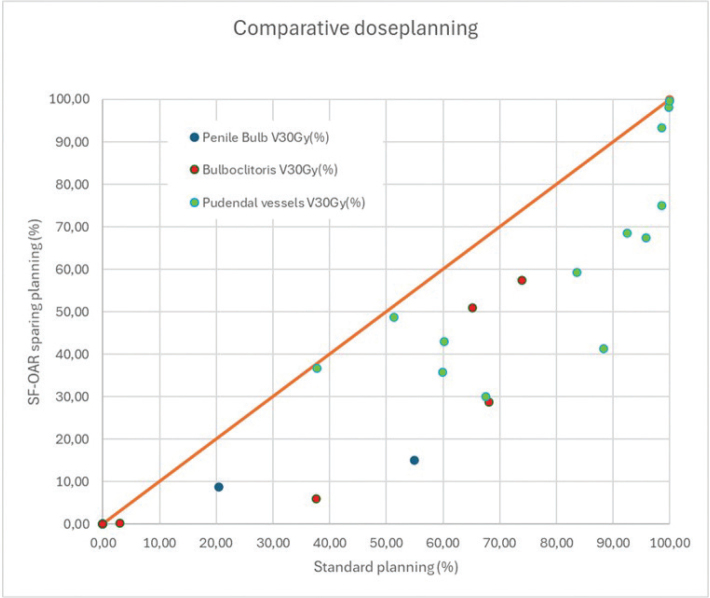
Scatter plot showing V30Gy (%) to pudendal vessels, penile bulb, and bulboclitoris with standard plans and SF-OAR (sexual function-related-organs at risk) sparing plans.

Dose to the inferior hypogastric plexus, while fully encompassed within the high-dose region in all plans (100% V30Gy and V40Gy), demonstrated only a small reduction in D2%, decreasing from 52.0 Gy in standard plans to 50.9 Gy in sparing plans (*p* = 0.0029). The pudendal vessels and nerves, including Alcock’s canal, showed significant dose reductions in V30Gy, which decreased from 90.6% to 67.4% (*p* < 0.001), and V40Gy, from 45.4% to 38.5% (*p* < 0.001). Only a minor change in D2% (51.3 Gy to 50.1 Gy, *p* = 0.021) was seen, reflecting that the cranial part of the volume was included in the target area (Table 2 and Figure 2).

In male patients, minor sparing of the neurovascular bundle was possible. Although D2% showed a decreasing trend, the difference was not significant. For the penile bulb, four of seven patients did not receive doses over 30 Gy in standard treatment plans. For the three patients with an initial dose to the penile bulb above 30 Gy, the SF-OAR-optimized plans resulted in a sparing of the organ (Figure 2).

Among female patients, dose sparing was primarily seen in the bulboclitoris. V30Gy was reduced from 63.6% in standard plans to 25.2% in SF-OAR-sparing plans (*p* = 0.015), and V40Gy decreased from 13.7% to 5.1% (*p* = 0.031). Although the D2% also dropped from 46.0 Gy to 41.6 Gy, this change did not reach statistical significance (*p* = 0.13) (Figure 2).

## Discussion and conclusion

With this study, we have developed a national consensus atlas for SF-OARs in an attempt to integrate sexual health preservation into dose planning of rectal cancer RT. Moreover, we have demonstrated that radiation dose to some SF-OARs can be reduced without compromising target coverage or standard OAR constraints. These results, with the identification of which SF-OAR that may be considered for organ-sparing, are warranted for future studies, aiming to investigate the impact of RT dose on sexual health in these patients.

For patients with rectal cancer, determining the impact caused by surgery versus that caused by RT is complex, but RT has been shown to be an independent risk factor for sexual dysfunction, as has the extent and type of surgery [1, 13]. In a cohort of patients treated with RT or chemoradiotherapy and observed by a watch and wait (WW) approach, there were no differences in sexual function when comparing WW- and TME (total mesorectal excision)-treated patients, except for a tendency toward more erectile dysfunction after RT and TME compared to RT alone. However, only 14% underwent TME in the cohort, and the absolute numbers or percentages cannot be extracted from the publication [14, 15]. A systematic review and meta-analysis found that RT did not have a negative impact on sexual dysfunction. However, the available data were limited by low quality and significant heterogeneity in both patient populations and treatment protocols. Notably, the authors emphasized that sexual dysfunction often emerges as a late-onset effect, which may not be adequately captured in studies focusing on early outcome measures [16].

Each of the delineated SF-OARs plays a distinct role in sexual physiology and could therefore be relevant for the development of radiation-induced sexual dysfunction. The inferior hypogastric plexus is a central autonomic structure that contributes to genital arousal, erection, and lubrication.

The pudendal nerves and vessels, traveling through Alcock’s canal, are essential for sensory and motor functions in the external genitalia and perineum and responsible for sensation of genital touch and pressure in both males and females. In males, the neurovascular bundle is critical for erectile function. The penile bulb has been associated with erectile outcomes in other studies, and while it does not seem to have a separate role, it is believed to be a surrogate for corpus cavernosum dose, which is important for penile rigidity during erection [17]. In females, the vagina, paracolpium, and bulboclitoris are involved in arousal, vaginal lubrication, and orgasm, but the exact pathophysiological mechanisms are less well-described [15]. Radiation to these structures may thus contribute to dryness, dyspareunia, and reduced sexual pleasure [18, 19]. One of the most frequently reported dysfunctions is vaginal dryness [20]. Vaginal lubrication is caused both by glandular secretion and by transudation of plasma from increased blood flow; therefore, the paracolpium was delineated as a separate structure to serve as a surrogate for vessels pertaining to this function [21].

Others have provided delineation guidelines for neurovascular bundle and pudendal arteries in males with prostate cancer [22, 23] both with focus on interrater agreement. Teunissen et al. also provided a comprehensive atlas for prostate cancer; our descriptions, however, differ as we focus on a standard rectal cancer target and description for both males and females. Furthermore, we describe the continuum from the inferior hypogastric plexus to the neurovascular bundle or paracolpium.

A detailed description of the bulboclitoris is also available for anal cancer radiotherapy planning. However, this does not include the entire structure – as part of corpus cavernosum/crus is left out [19]. Definition of the vagina is available from [24] but does not separate paracolpium from muscular vaginal wall. Delineating the full structure of bulboclitoris and separating the muscular vagina from the paracolpium will hopefully improve coupling these structures to clinical outcomes.

Erectile dysfunction after radiotherapy for prostate cancer and radiation dose to the penile structures have been described in a systematic review, including data on penile bulb and vascular structures, such as neurovascular bundle and internal pudendal arteries. As reviewed, sparing of especially the penile bulb has been studied for prostate cancer. The results are conflicting, and studies on vascular sparing are only just emerging, but no definitive constraints have been defined. However, for the penile bulb, mean dose < 20 Gy has been suggested [11]. Direct comparison to this study is difficult, as radiation dose and volume differ, as do other treatments such as anti-androgen therapy and surgery.

Both radiation dose and irradiated volume increase the risk of vaginal stenoses after radiotherapy [10]. It is primarily studied in gynecological cancers, but a study on rectal and anal cancer found a relation to several dose levels, especially mean dose > 43 Gy resulting in increased risk of stenoses [25]. The potential of sparing the bulboclitoris has been shown for anal cancer dose planning but with no clinical data [19]. The magnitude of sparing is difficult to compare directly with our data due to differences in target volume. Sparing of pudendal arteries, plexus hypogastricus, and paracolpium has not previously been described or investigated for females.

It is important to note, although not addressed in this study, that radiation exposure to ovaries and testes can result in hormonal dysfunction and contribute to post-treatment sexual dysfunction. Such effects can, however, more easily be assessed by routine blood screening and can to some degree be managed by hormonal replacement therapy.

Our findings confirm that certain SF-OARs, particularly those outside high-dose regions like the pudendal vessels, bulboclitoris, and penile bulb, can be spared consistently, within standard IMRT/VMAT workflows. Selectively including these relevant SF-OARs could be feasible for RT planning. For other SF-OARs, the optimization primarily resulted in lowering maximum doses, but inhomogeneous target coverage, still fulfilling optimization criteria, could perhaps result in even lower doses also for these structures. However, gaining knowledge on dose effect relations of the SF-OARs, which cannot be spared, would also provide new insights essential for general understanding of sexual dysfunction and patient information.

The next phase of this research will focus on implementing a detailed, symptom-specific sexual function questionnaire and correlating radiation dose to individual SF-OARs with specific domains of sexual dysfunction, also including patients’ perspectives. This approach will enable the development of dose constraints tailored to protecting sexual function and guide future patient-centered RT planning across pelvic malignancies.

This planning study is limited by its small cohort of patients without data on clinical outcome. Nonetheless, it establishes a foundation for future prospective trials aimed at optimizing RT planning for rectal cancer. Moreover, the atlas may be applicable to other pelvic malignancies, including gynecologic and urologic cancers, where similar dysfunctions are observed. However, it should be kept in mind that the described anatomy can be the subject to change and displacement based on tumor localization, and that the precise SF-OAR sparing potential also differs based on the defined target volumes for the different diagnoses. We used an MRI in treatment position. A diagnostic MRI can probably aid in delineation of SF-OAR, but we have not verified this. Identifying these structures by CT only would be challenging for most SF-OAR.

In conclusion, we developed a consensus-based SF-OAR atlas for rectal cancer RT and demonstrated that several structures can be spared without compromising oncologic objectives. Through the continued work, we aim to support patient-centered radiotherapy planning that prioritizes long-term sexual health and quality of life.

## Supplementary Material



## Data Availability

The data supporting this study are not publicly available due to National legislation. Access may be granted by the corresponding author upon reasonable request and with appropriate approvals.
