# Supplementary material for: Defining and sparing sexual function-related organs at risk for rectal cancer radiotherapy
Source: Acta Oncol. 2025 Aug 28;64:44011. doi: 10.2340/1651-226X.2025.44011 (PMC12406747; doi:10.2340/1651-226X.2025.44011)
Supplement: Supplementary file 1 [file AO-64-44011-s1.pdf]

Supplementary material has been published as submitted. It has not been copyedited, or typeset by Acta Oncologica

Summary of the Danish Dose Planning Guideline for Rectal Cancer.

Patients were positioned supine on a flat tabletop for both imaging and treatment. For an optimal and reproducible positioning suitable immobilization aids including knee cushion and when necessary, foot support, was used. A CT scan was acquired in the treatment position. For MRI based delineation an MR scan was acquired in the same position as for CT and treatment to ensure an optimal image registration and anatomical alignment for accurate delineation of target volumes and organs at risk (OARs). A MR only workflow using synthetical CTs can be used if implemented in the clinic (one center for this study). For sexual function-related organs at risk (SF-OARs) an atlas was developed as a supplementary to the national guideline.
Dose plans used either up to 9-12 field (Ethos) IMRT and 1-3 arcs VMAT. Prescribed dose was 50.4 Gy in 28 fractions.

Target volumes:
Target volumes were delineated according to national rectal cancers radiotherapy guidelines.
CTV-T (Tumor Clinical Target Volume):

- Encompassed the gross tumor volume (GTV-T) with a 5 mm margin radially and a 10 mm margin cranio-caudally to account for microscopic spread and regions at high risk for microscopic spread (e.g., mesorectum, presacral space, and posterior bladder wall).

CTV-E (Elective Clinical Target Volume):

- Included the elective nodal regions, standard presacral, mesorectal and lateral pelvic nodes. External iliacal nodes, ischiorectal fossa and ingvinal lymphnodes included on individual basis.

ITV-T (Tumor Internal Target volume):

- Was generated for CTV-T by adding the following margins to account for organ motion:
    - 7 mm laterally and cranio-caudally
    - 4 mm posteriorly
    - 10 mm anteriorly

ITV-E (Elective Internal Target volume):

- Was generated for CTV-E adding an anteriorly margin of 5-20 mm.

PTV-T/E (Tumor/Elective Planning Target Volume):

- Were generated by adding locally defined uniform margins to the ITV-T/E, respectively to account for setup uncertainties and internal organ motion.

Dose-volume objectives for target volumes followed national guidelines:
Standard dosimetric nomenclature is applied to describe target coverage and OAR constraints. Here, VxGy refers to the volume (in cm^3 or as a percentage) receiving at least x Gy, while Dx% denotes the minimum dose received by x% of the volume, expressed as a percentage of the prescribed dose (50.4 Gy).

- CTV-T and CTV-E:
    - V95% = 100%
- PTV-T:
    - V95% ≥ 99%
    - V90% = 100%

- $V105\% \leq 1\%$
- PTV-E:
  - $V95\% \geq 98\%$
  - $V90\% = 100\%$
  - $V105\% \leq 1\%$
- Further: $D2\% \leq 107\%$ (dose to the hottest 2% of the volume was kept below 107% of the prescribed dose.

These criteria ensured target coverage of both the high risk and the elective areas.

Organs at risk constraints:
In addition to target volume coverage, dose constraints for standard organs at risk were applied in accordance with national guidelines:

- Bowel bag:
  - Volume receiving ≥45 Gy: V45Gy < 300 cm^3
  - Volume receiving ≥30 Gy: V30Gy < 600 cm^3

- Bladder:
  - Volume receiving ≥50 Gy: V50Gy < 20%
  - Volume receiving ≥35 Gy: V35Gy < 75%

- Femoral heads, sacrum, sacroiliac joints, and penile bulb:
  - The volume of these structures receiving 50 Gy was minimized as much as possible, with no specific threshold, but kept to the lowest achievable levels.

PTV target coverage was prioritized in all planning scenarios. SF-OAR sparing was pursued only when the target volume coverage criteria were met without compromise and the standard OAR constraints were adhered to.

Atlas for SF-OAR

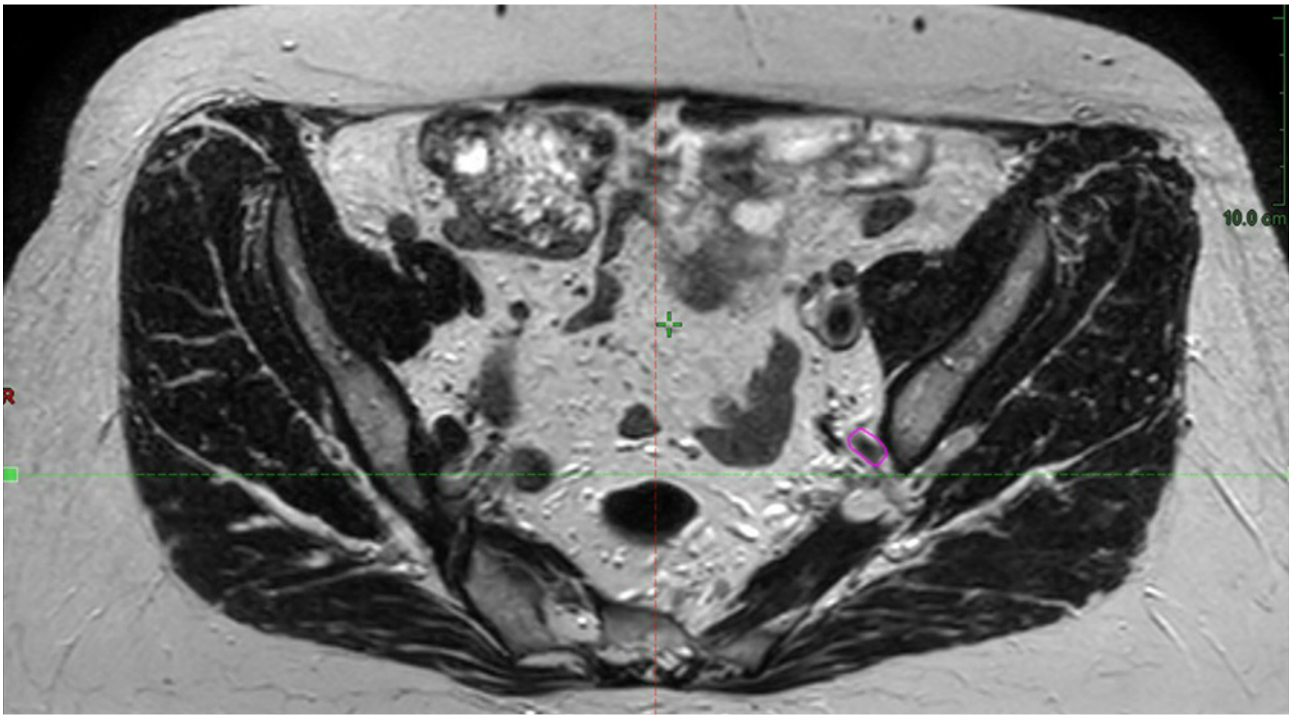

1. F. Pudendal vessel (magenta) where it origins from internal iliacal vessels (L). On the right side it has not left the internal iliacal vessels.

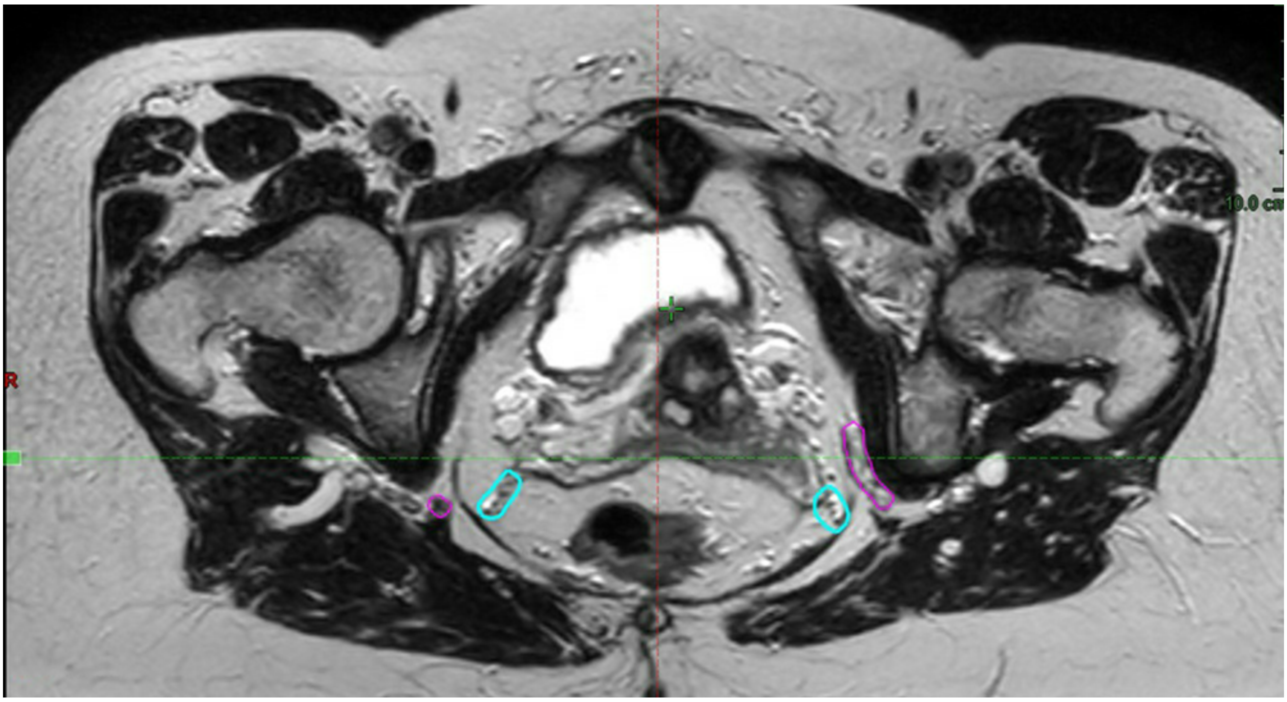

2. F. Pudendal vessel (magenta) on the left side it has just re-entered the pelvis to run along obturator internus muscle in Alcock´s canal. On the right side the pudendal vessel has just circumvented the ischial spine before re-entering the pelvis.
   The inferior hypogastric plexus (cyan) where vascular ingress is seen in the mesorectal fascia.

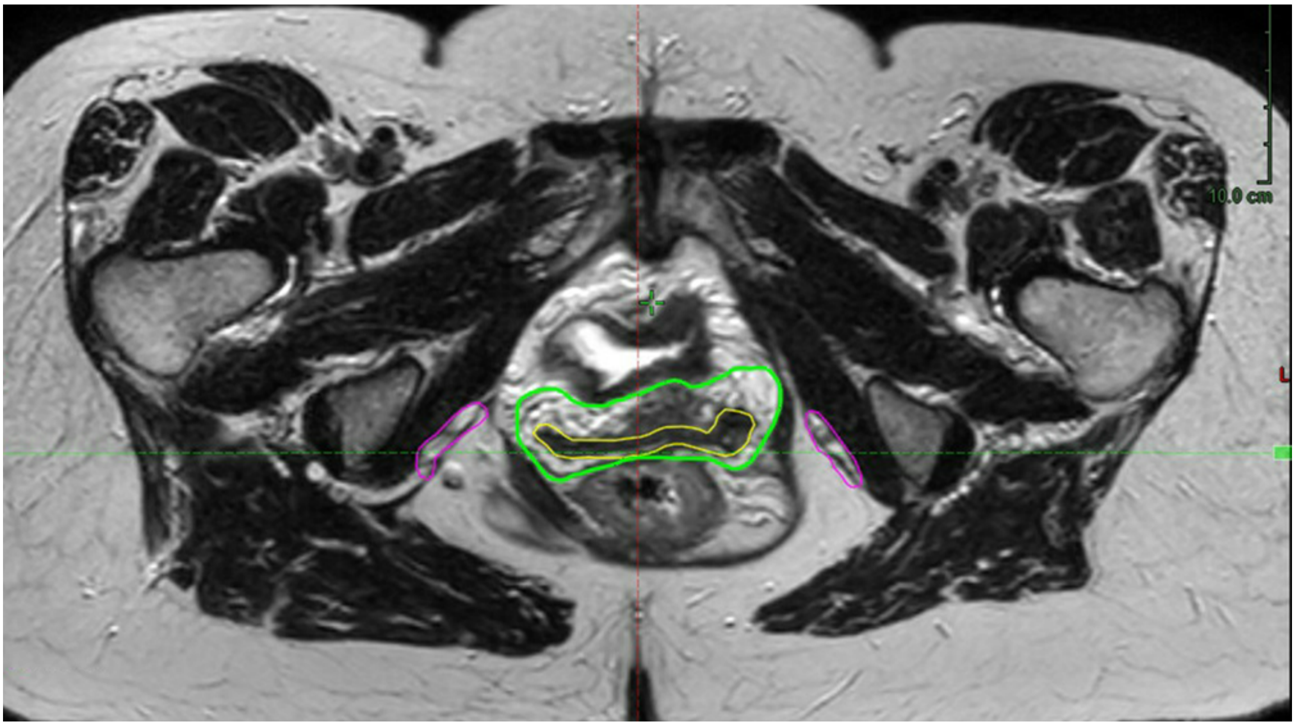

3. F. The pudendal vessels (magenta) runs anteriorly in Alcock´s canal. The muscular vaginal wall (yellow) is encompassed by the paracolpium (green).

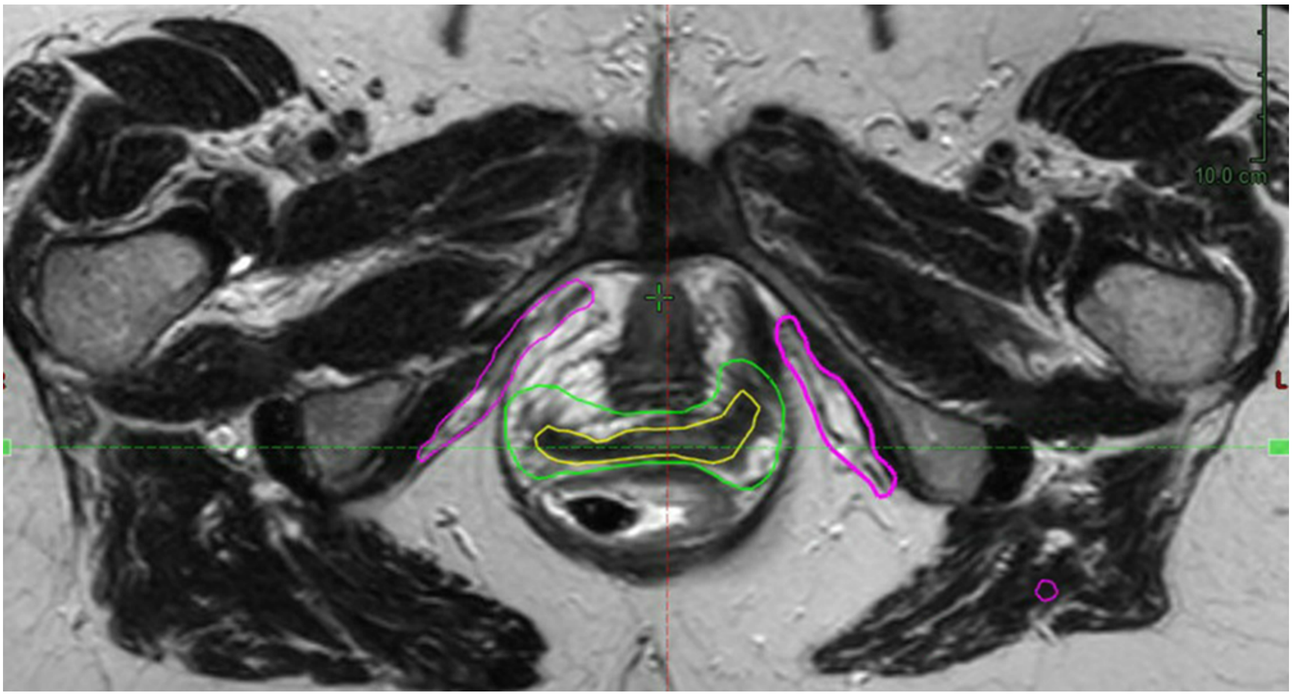

4. F. The pudendal vessels (magenta) runs anteriorly in Alcock´s canal. The muscular vaginal wall (yellow) is encompassed by the paracolpium (green).

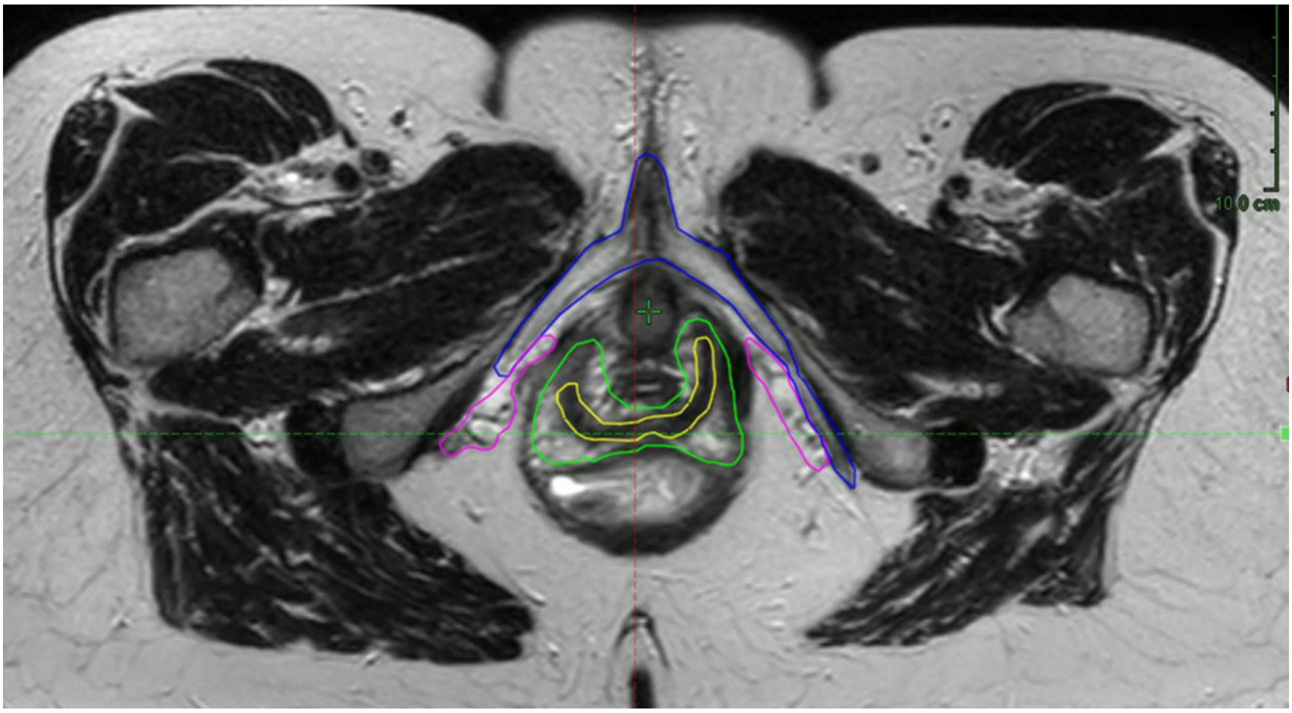

5. F. The pudendal vessels (magenta) runs anteriorly in Alcock´s canal. The muscular vaginal wall (yellow) is encompassed by the paracolpium (green). The crura of the bulboclitoris (blue) runs along the ischiopubic rami bilaterally.

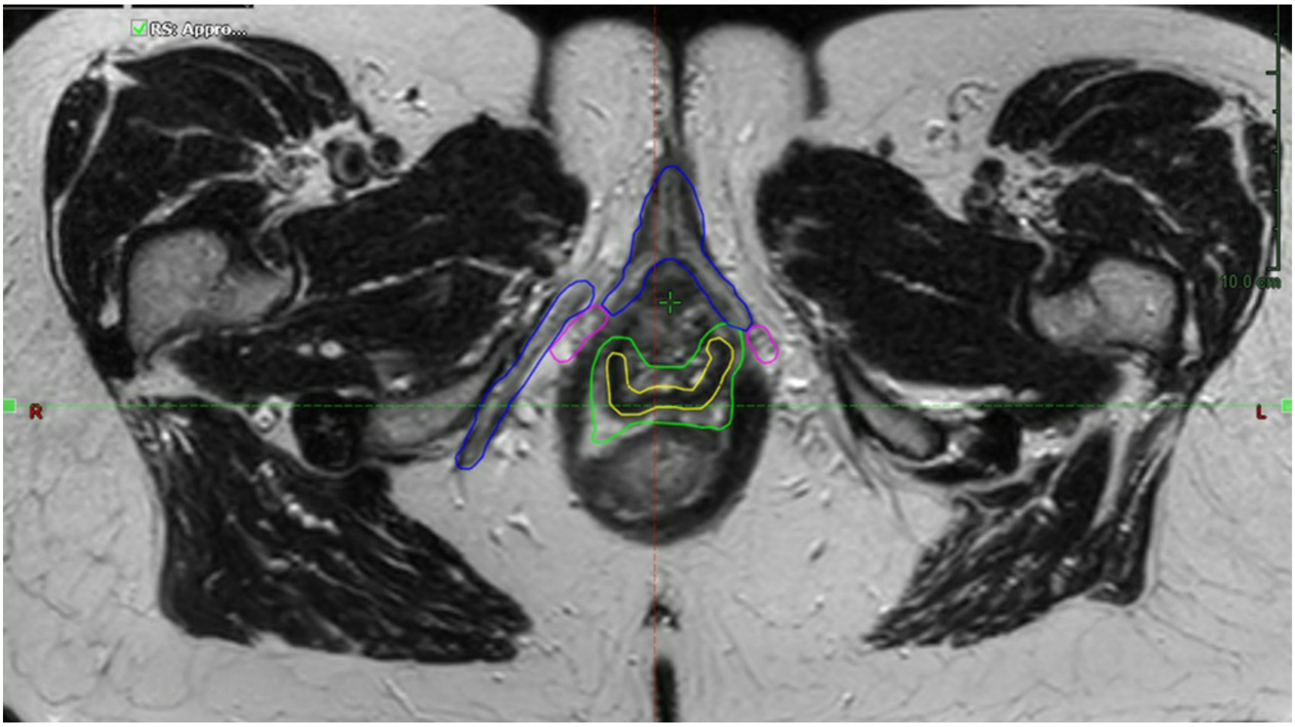

6. F. The pudendal vessels (magenta) end anteriorly in the bulb of the bulboclitoris (blue). The crura is still visible on the right side.
The muscular vaginal wall (yellow) is encompassed by the paracolpium (green).

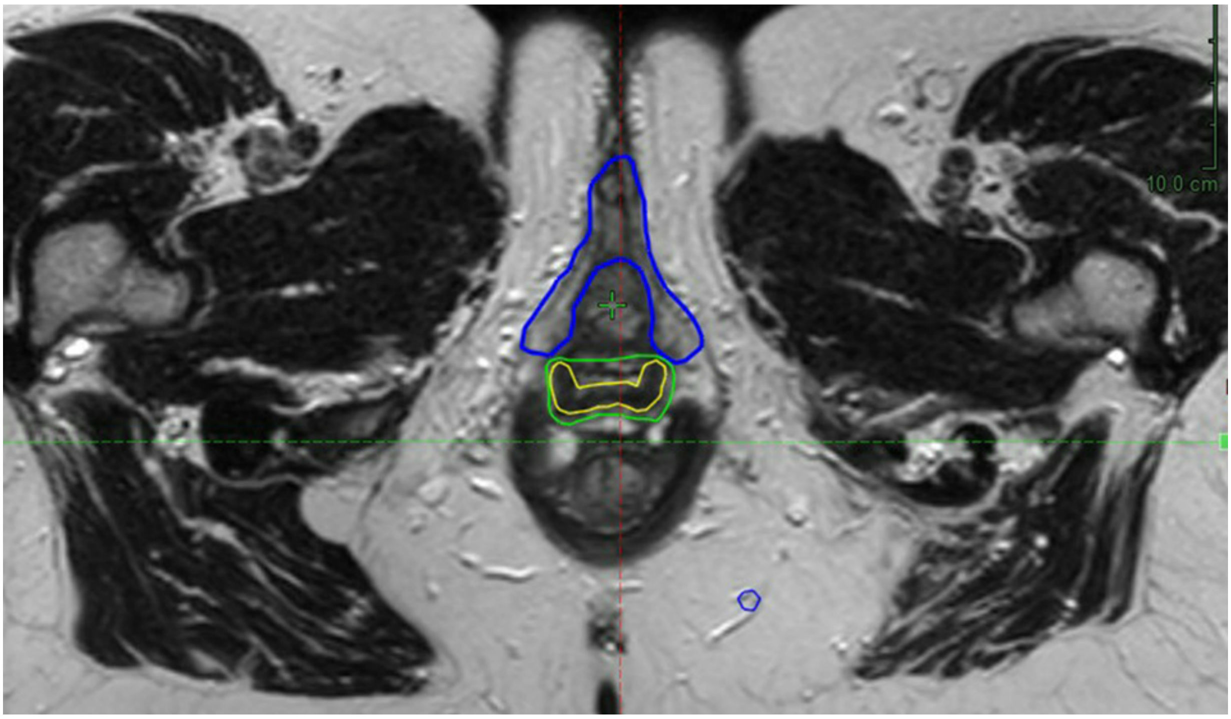

7. F. The bulb of the bulboclitoris (blue) ends posteriorly at the anterior paracolpium (green).

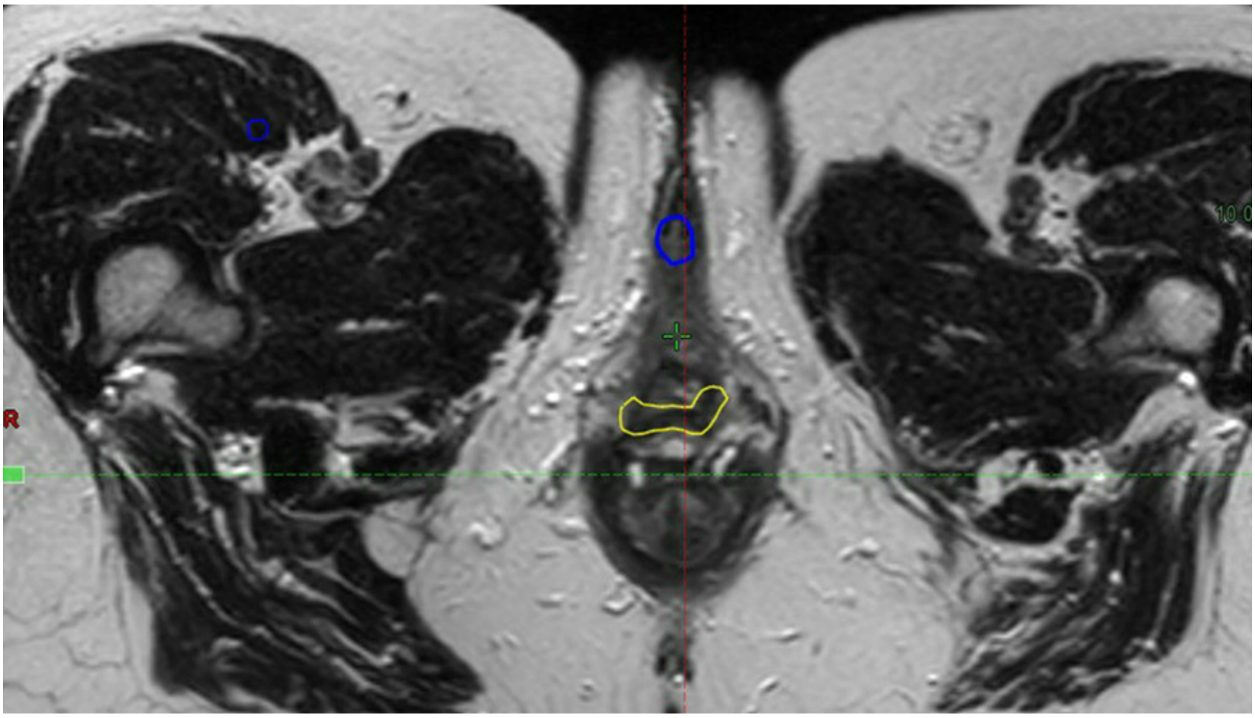

8. F. The glans of the bulboclitoris (blue) continues after disappearance of the bulbus. At the most inferior slice the muscular vagina (yellow) is not encompassed by visible paracolpium.

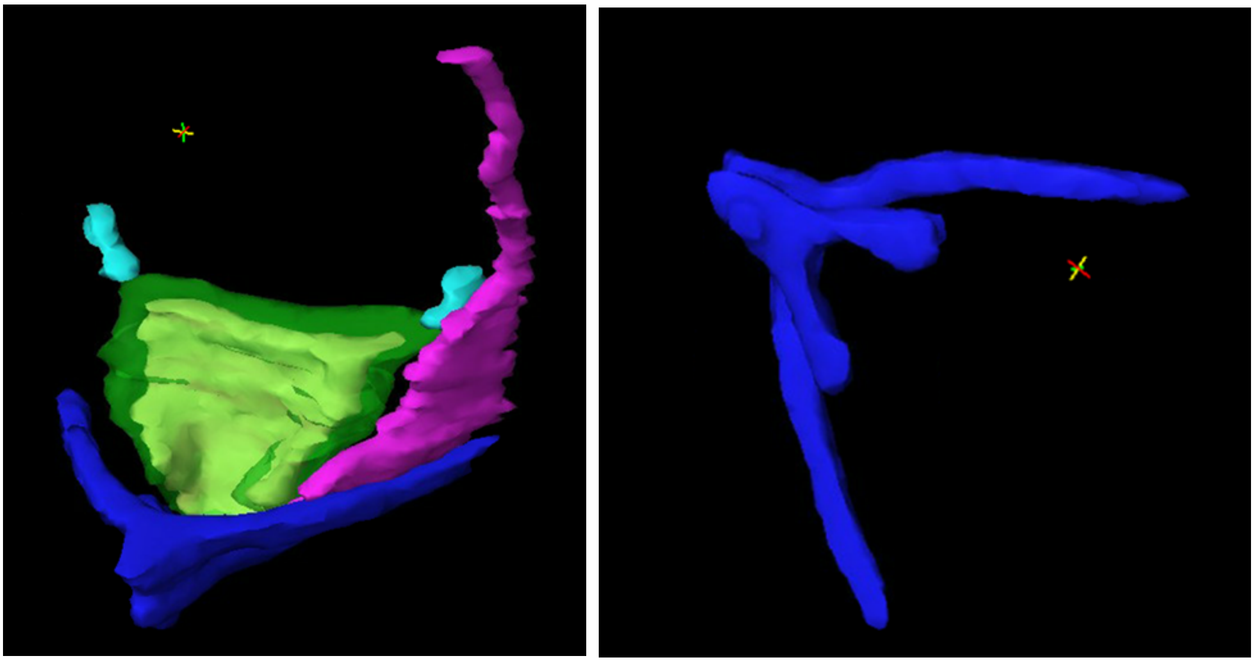

9. F. 3D visualization of relations of pudendal vessels/Alcock´s canal (left)(magenta), the inferior hypogastric plexus (cyan), the vagina (yellow) encompassed by the paracolpium (transparent green). The bulboclitoris including crura (blue) is also visualized from an inferior aspect showing extensions of the glans, the bulb and the crura.

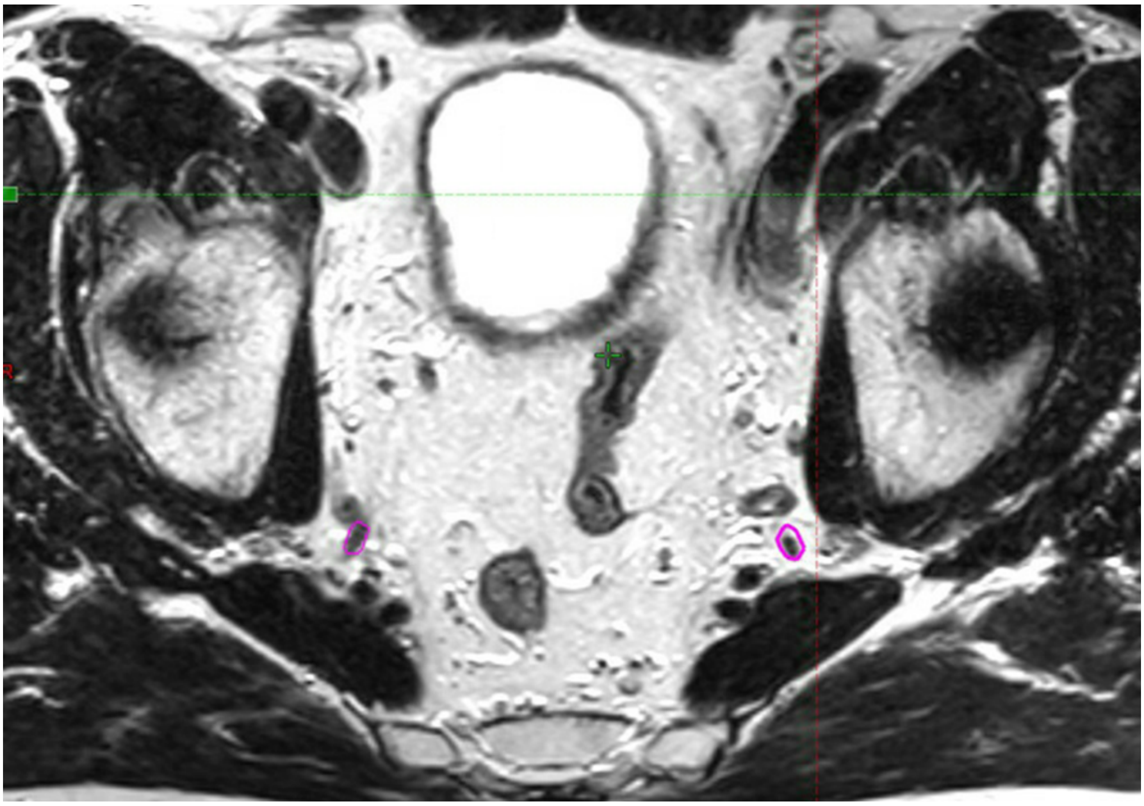

1. M. Pudendal vessel (magenta) where it origins from internal iliacal vessels.

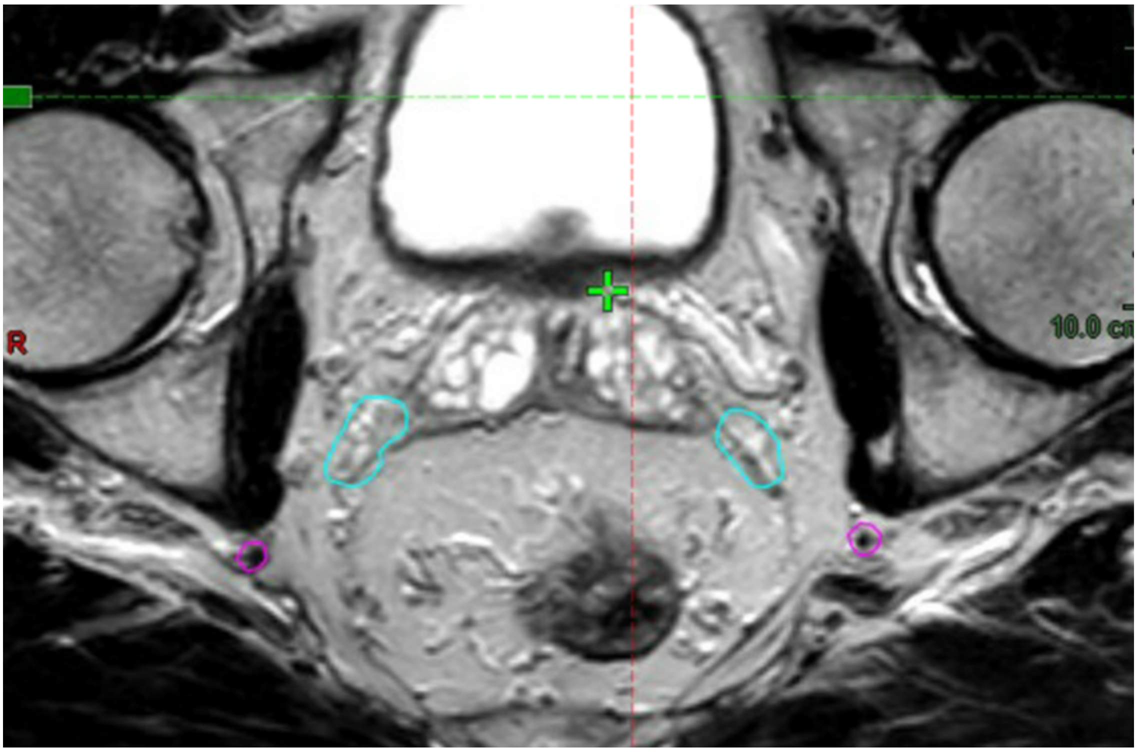

2. M. Pudendal vessel (magenta) are exiting the pelvis bilaterally.
   The inferior hypogastric plexus (cyan) where vascular ingress is seen in the mesorectal fascia.

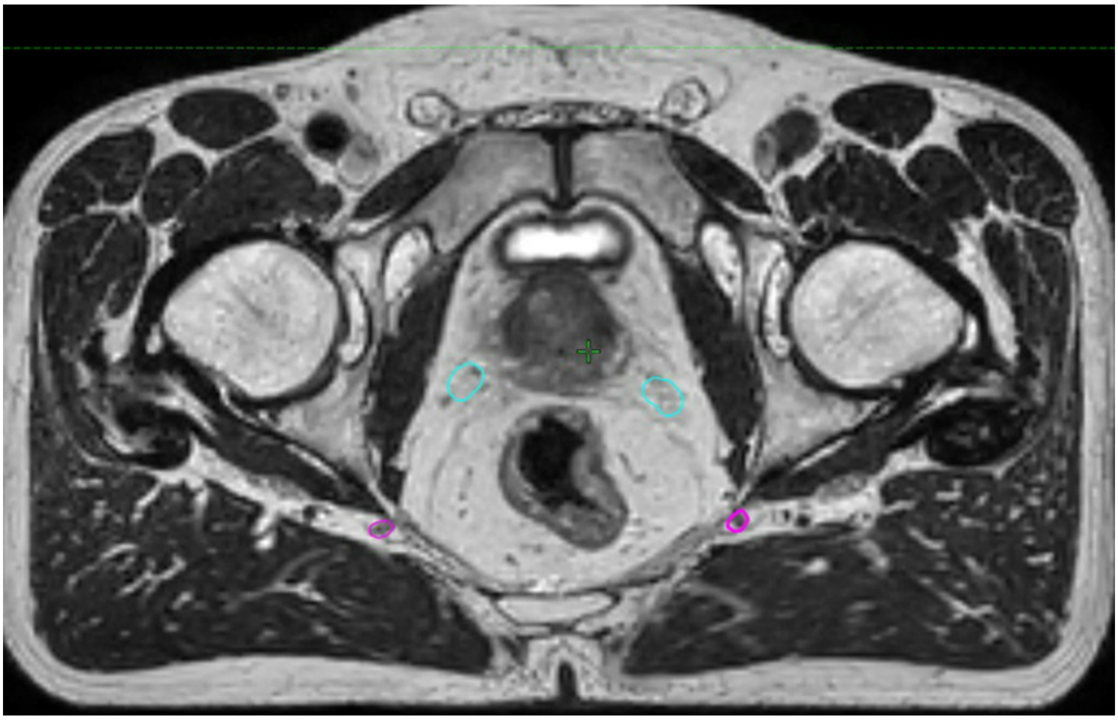

3. M. Pudendal vessel (magenta) before they circumvent the ischial spine and reenter the pelvis.
   The inferior hypogastric plexus (cyan) at the last slice before they end in the neurovascular bundles.

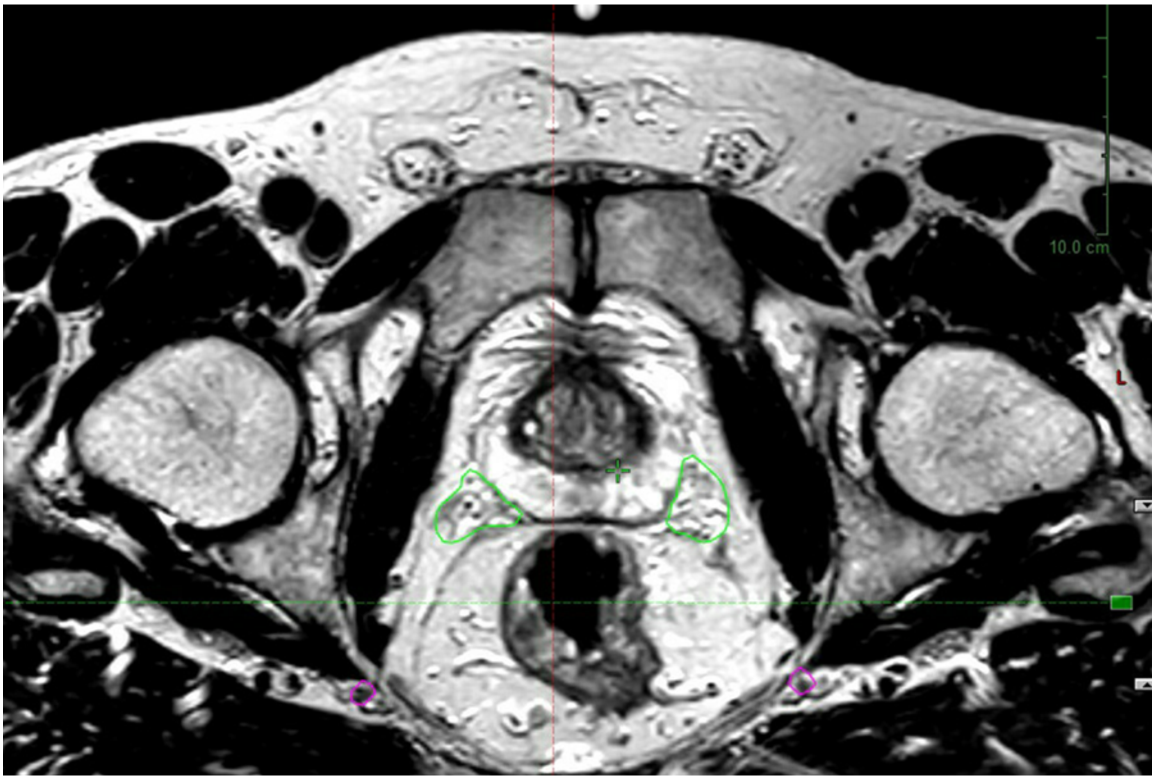

4. M. Pudendal vessel (magenta) before they circumvent the ischial spine and reenter the pelvis. Most cranial slice with neurovascular bundle (green).

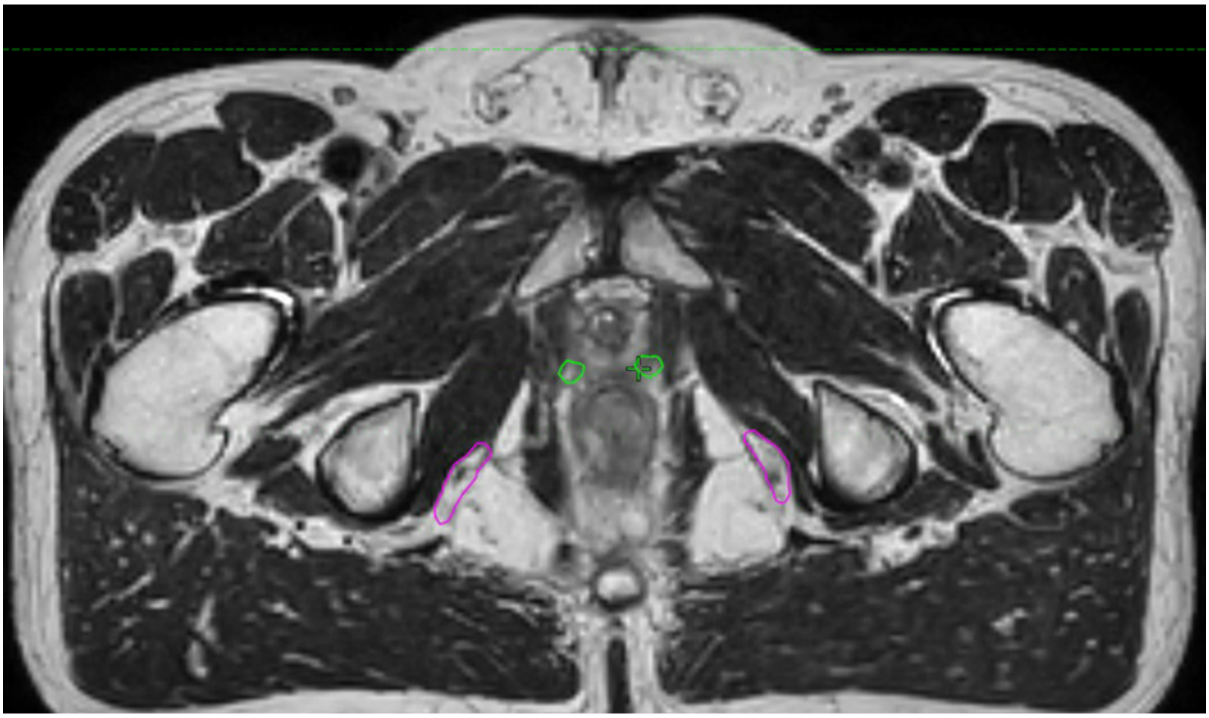

5. M. The pudendal vessels (magenta) runs anteriorly in Alcock´s canal. The most caudal slice with visible neurovascular bundle subjacent to the prostate apex (green).

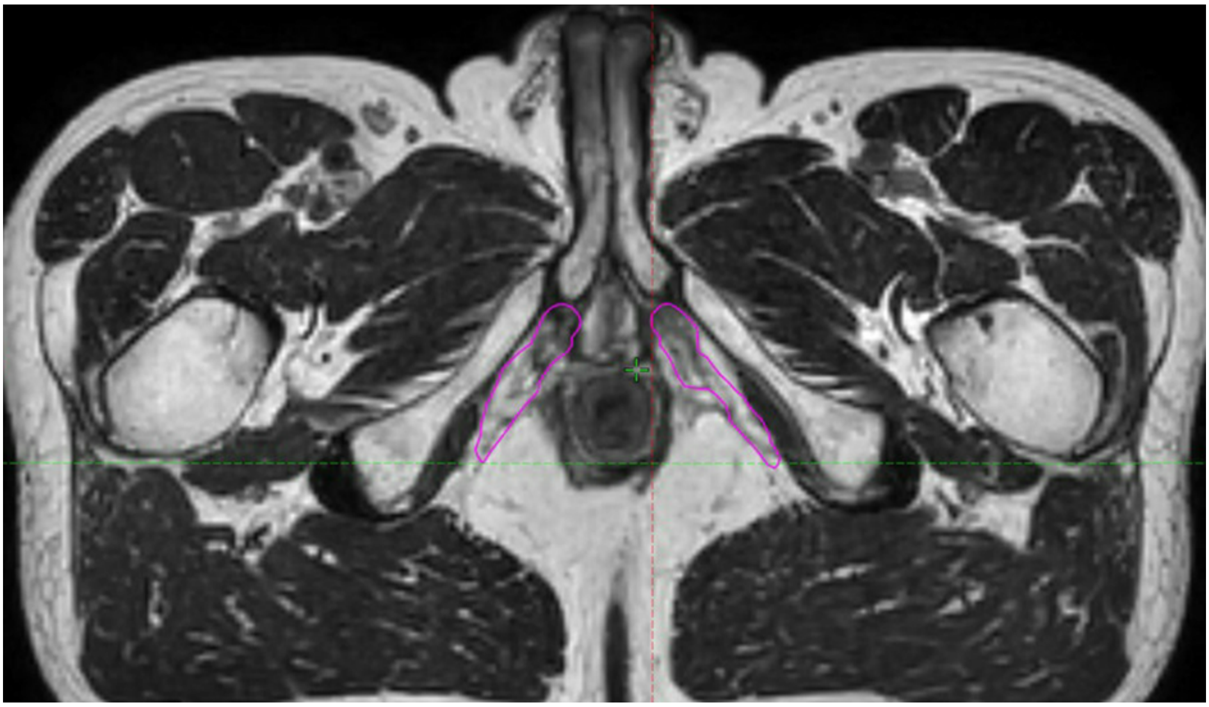

6. M. The pudendal vessels (magenta) end anteriorly in the root of the penile structures.

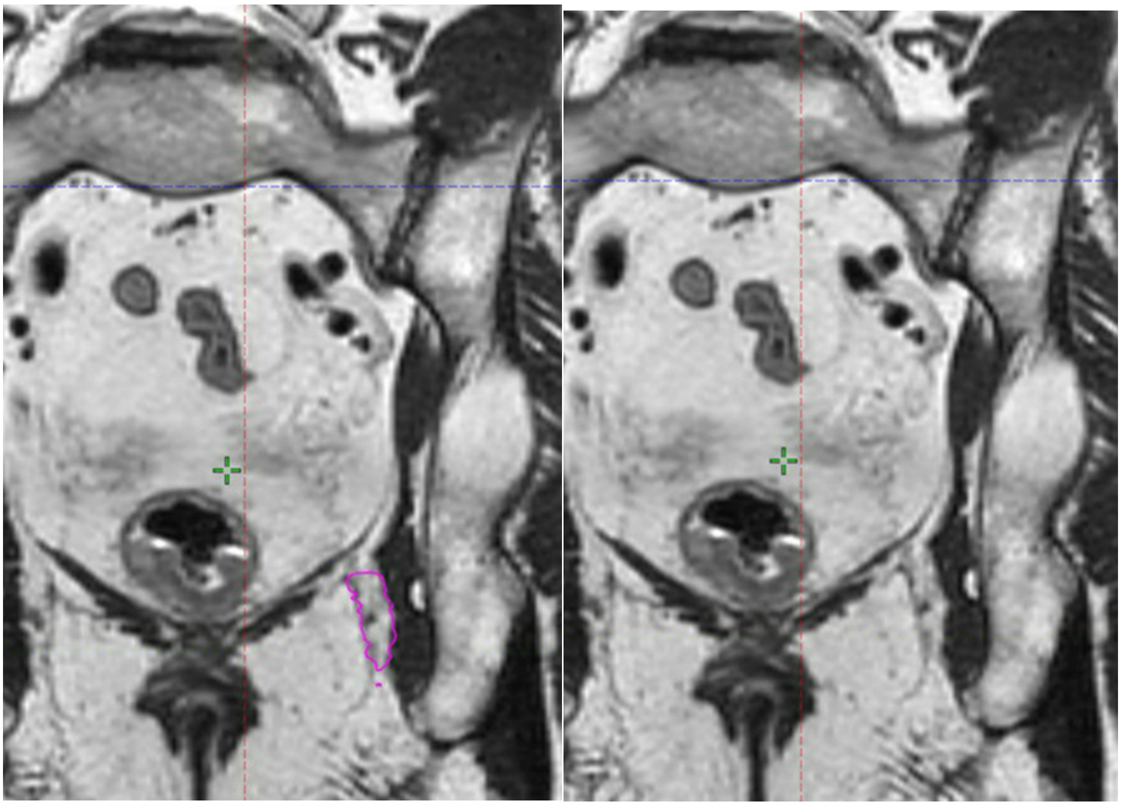

7. M. Coronal slice of the delineated (magenta) and un-delineated alcock´s canal with pudendal vessels.

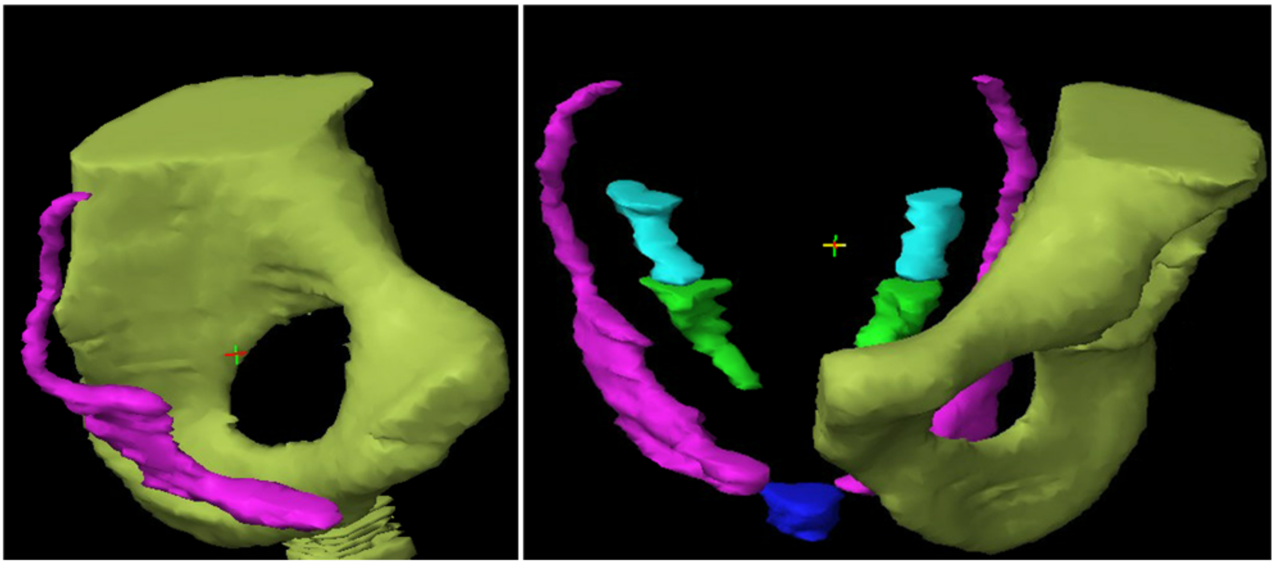

8. M. 3D visualization of the relations of pudendal vessels/Alcock´s canal (magenta) as it exits the pelvis circumvent the ischial spine and reenter the pelvis to run anteriorly in Alcock´s canal, the inferior hypogastric plexus (cyan), where in converges with the neurovascular bundle (green) The penile bulb (blue) is visualized where the anterior part of the pudendal vessels end.

Descriptive table SF-OAR

OAR	M/F	Description
A./N. pudendae + Alcock´s canal	M/F	The pudendal nerve demonstrates a close anatomical relationship with the pudendal artery. Due to the inherent difficulty in directly visualizing the nerve on imaging modalities, a delineation strategy involving a 5mm contour surrounding the arterial lumen is recommended.<br>The cranial extent of this delineation is defined by the origin of the pudendal artery from the internal iliac vessels. The nerve and artery follow a trajectory involving exit from the pelvic cavity, circumvention of the ischial spine, re-entry into the pelvis, and a course along the obturator internus muscle. Within Alcock's canal, which is best visualized in the coronal plane as a discontinuity in the muscular fascia, comprehensive delineation of the canal is indicated. The nerve and artery then extend in an anterior direction, positioned bilaterally to the anal canal and subsequently the urethra. Termination differs based on sex: the root of the penis in males, and a path medial to the clitoral crura culminating at the clitoral bulb in females. The caudal boundary is established by the urogenital diaphragm (21).
Inferior hypogastric plexus.	M/F	The inferior hypogastric plexus is anatomically localized at the periphery of the mesorectum, specifically at the sites of vascular ingress. On axial imaging, its typical presentation is at the 10 and 2 o'clock positions, with a craniocaudal placement generally within the mid-mesorectal region. The plexus is clearly delineated at the mesorectal border prior to its anterior extension. Its dimensions are reported as 15-40 mm along the craniocaudal axis and 10-30 mm along the anteroposterior axis.<br>In the male, the caudal and anterior portions of the plexus exhibit a confluence with the neurovascular bundle. This point of integration corresponds to the adherence of the neurovascular bundle to the external aspect of the prostate gland.<br>In the female, the position of the plexus may exhibit increased posterior and inferior displacement, contingent upon the degree of uterine flexion. Its distal termination occurs within the vascular components of the paracolpium.
Neurovascular bundt	M	The neurovascular bundle is located in intimate association with the posterolateral aspect of the prostate gland. Its cranial origin is at the termination of the inferior hypogastric plexus or at the prostatic base. Subsequently, it traverses the posterior surface of the prostate and proceeds in an inferior direction, situated subjacent to the prostatic apex (21).
Penile Bulb	M	As penile bulb was a part of standard delineation we refer to our national standard for delineation of this (25)
Vagina	F	The vagina is a central, muscular tube, approximately 7-9 cm in length, connecting the cervix or vaginal vault to the vulva. Its wall is composed of three layers: mucosa, muscle, and adventitia. MRI T2-weighted imaging is necessary to visualize the high-signal mucosa and secretions, which contrast with the low-signal muscle layer. In premenopausal women, the vagina's anterior and posterior walls are coated by surrounding pelvic structures, resulting in an H-shaped or W-shaped appearance on axial MRI. However, postmenopausal women, experiencing a lack of hormonal stimulation, show a decrease in vaginal wall thickness, a loss of distinguishable mucosal rugae, and less clear separation of wall layers on MRI. (26).<br>The vagina's superior limit is the cervix or vaginal vault, and its inferior limit is the slice just before the vulva (27). The position of

		the inferior limit may also be defined by a line that extend from the inferior pubis symphysis to the meatus urethra externus.
Paracolpium	F	The paracolpium is composed of vessels and connective tissue encircling the vagina, the structure is most easily identified on axial T2-weighted sequences. On these images, it presents as a hyperintense region, measuring 3-10 mm in thickness, that encompasses the hypointense muscular wall of the vagina (26, 27). Its anatomical position is posterior to the urethra. Posterior boundary is the mesorectal fascia and more caudally the sphincter apparatus. The cranial boundary is defined by the termination of the muscular vagina, while the caudal extent is observed in the slices immediately cranial to the introitus, ceasing where the hyperintense tissue disappears.
Bulboclitoris	F	The bulboclitoris, an erectile structure, is defined by its boundaries relative to surrounding tissues and its components. Cranially, it is bounded by the suspensory ligament (to the pubic arch), the inferior ischiopubic rami (superior to crura), and the perineal membrane (superior to bulbs). Caudally, the crura are covered by ischiocavernosus muscles and the bulbs by bulbospongiosus muscles, both surrounded by fat; the glans is the most inferior and anterior projection. Anteriorly, the bulbs merge with the corpus forming the glans, with surrounding fat. Posteriorly, the crura taper along the ischiopubic rami, bounded by fat and the pudendal vessels; the posterior crura diverge laterally from the bulbs. The posterior border of the bulbs is in line with the anterior part of the paracolpium. lateral edge of the bulbs is bounded by the medial crura, with clearer demarcation posteriorly due to diverging crura and fat. Medially, the crura and bulbs are often indistinguishable anteriorly, but distinguished posteriorly by fat; the medial bulbs surround the urethral and vaginal openings(18).

Where relevant, references to other publications on delineation of these structures are noted in the table. However, our definitions vary to some degree as we provide definitions related to a rectal target and both for M and F.

Dose volume histograms for Bladder and Bowel bag.

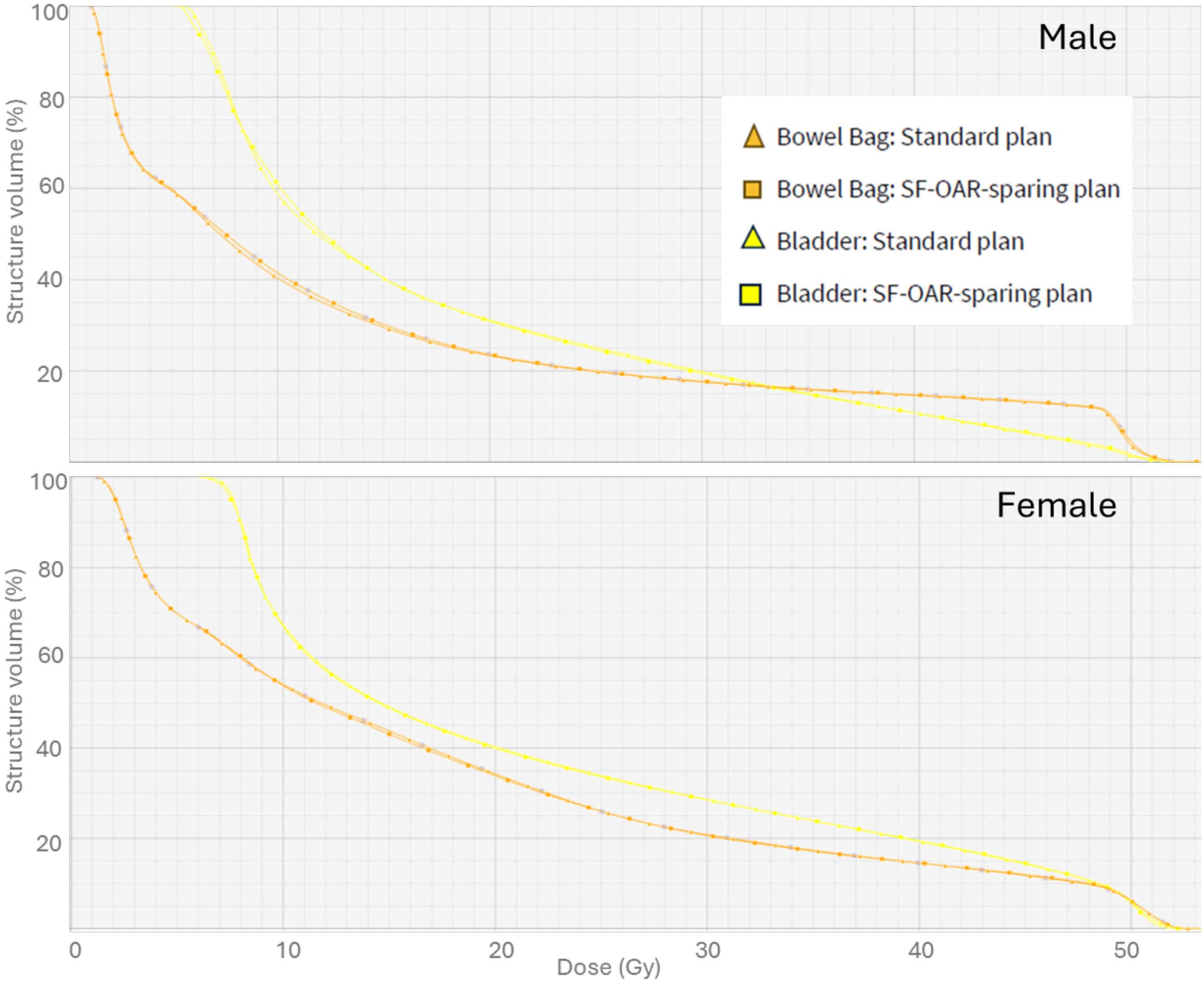

Supplementary figure 1. Representative dose–volume histograms for bowel and bladder, comparing standard treatment plans (solid lines) with SF-OAR–sparing plans (dashed lines).
